# Draft Genome Sequence of “*Candidatus* Phytoplasma asteris” Strain OY-V, an Unculturable Plant-Pathogenic Bacterium

**DOI:** 10.1128/genomeA.00944-14

**Published:** 2014-09-25

**Authors:** Shigeyuki Kakizawa, Ayaka Makino, Yoshiko Ishii, Hideyuki Tamaki, Yoichi Kamagata

**Affiliations:** Bioproduction Research Institute, National Institute of Advanced Industrial Science and Technology, Tsukuba, Ibaraki, Japan

## Abstract

Phytoplasmas are unculturable plant-pathogenic bacteria causing devastating damage to agricultural production worldwide. Here, we report the draft genome sequence of “*Candidatus* Phytoplasma asteris” strain OY-V. Most of the known virulence factors and host-interacting proteins were conserved in OY-V. This genome furthers our understanding of genetic diversity and pathogenicity of phytoplasmas.

## GENOME ANNOUNCEMENT

Phytoplasmas are plant-pathogenic bacteria in the class *Mollicutes*. They are transmitted by insect vectors and infect over 700 plant species, causing devastating damage to agricultural production worldwide. Infected plants show a wide range of symptoms, including dwarfism, yellowing, witches’ broom, and phyllody (1). Despite the agricultural importance and unique features of phytoplasmas, the difficulty of *in vitro* culture has hindered their molecular characterization. Genome sequences of several phytoplasma strains have been reported recently, and they have enabled us to better understand the molecular mechanism of the virulence of phytoplasmas. However, the number of available phytoplasma genomes is still limited, so more information would be needed to further understand the pathogen. Here, we sequenced the draft genome of “*Candidatus* Phytoplasma asteris” strain OY-V, which is derived from strain OY-W (2) and known to produce severe symptoms. A genome of strain OY-M causing mild symptoms was previously sequenced (3). Thus, the OY-V genome was expected to provide novel insights into the molecular basis for its pathogenicity.

Phytoplasma cells were collected from phytoplasma-infected plant tissues (garland chrysanthemum) by a serial centrifugation method (2). Plus-field gel electrophoresis was performed and a genomic band (approx. 1 Mbp) was excised from the gels. DNA was amplified by GenomiPhi (GE Healthcare) and then sequenced using Illumina Hi-Seq2000 (100-bp paired-end) and 454 GS FLX with a 3-kbp mate-pair library (400-bp paired-end). Hi-Seq2000 and GS FLX generated 34,111,275 reads (3,381,554,184 bp) and 275,443 reads (123,471,699 bp), respectively. Sequence assembly was performed by Velvet version 2.0 and GS *de novo* assembler version 2.8, and resulted in 843 contigs (1,389,296 bp). Among them, 170 contigs (739,609 bp) were identified as phytoplasma-derived by BLAST analysis. These contigs were automatically annotated using the Microbial Genome Annotation Pipeline (4) and deposited to DDBJ. All reads were remapped to the final 170 contigs, and 77.3% of the reads were derived from phytoplasma. The other 22.7% of the reads were derived from plant chromosomes, organelles, fungi, endophytic bacteria, etc. The average coverage was 3,648-fold. Even though we sequenced a lot, most of the possible mobile unit (PMU) regions could not be obtained, possibly because of high repetition (3, 5).

The OY-V contings, with a G+C content of 27.5%, contained 920 CDSs and 27 tRNAs. Most of the known virulence factors and host-interacting proteins were also conserved in the OY-V genome, and several secreted proteins were identified. Homologous genes of TENGU (pathogenic factor of witches’ broom and dwarfism; PAM765) (6), SAP11 (another pathogenic factor of witches’ broom; AYWB370; PAM577) (7), Amp (antigenic membrane protein that interacts with insect microfilament; PAM122) (8), and Imp (immunodominant membrane protein conserved in most phytoplasmas; PAM610) (9) were identified. Neither SAP54 (pathogenic factor of phyllody; AYWB224; PAM049) (10) nor IdpA (immunodominant membrane protein in western X-disease phytoplasma group) (11) homologue was found. Similar to other phytoplasmas, there were no F-type ATPase component genes in OY-V. Intact genes for folate biosynthesis (*folP* and *folK*) were conserved in OY-V, although the strain-specific decay of the genes was previously reported (12).

The annotated genomic sequence would further our understanding of the genetic diversity of phytoplasmas and provide insights into the molecular basis for their pathogenicity.

### Nucleotide sequence accession number.

This whole-genome shotgun project has been deposited in DDBJ/ENA/GenBank under the accession number BBIY00000000.
